# Long short-term memory-based deep learning model for the discovery of antimicrobial peptides targeting *Mycobacterium tuberculosis*

**DOI:** 10.1093/bioadv/vbaf274

**Published:** 2025-10-31

**Authors:** Linfeng Wang, Susana Campino, Taane G Clark, Jody E Phelan

**Affiliations:** Faculty of Infectious and Tropical Diseases, London School of Hygiene and Tropical Medicine, London WC1E 7HT, United Kingdom; Faculty of Infectious and Tropical Diseases, London School of Hygiene and Tropical Medicine, London WC1E 7HT, United Kingdom; Faculty of Infectious and Tropical Diseases, London School of Hygiene and Tropical Medicine, London WC1E 7HT, United Kingdom; Faculty of Epidemiology and Population Health, London School of Hygiene and Tropical Medicine, London WC1E 7HT, United Kingdom; Faculty of Infectious and Tropical Diseases, London School of Hygiene and Tropical Medicine, London WC1E 7HT, United Kingdom

## Abstract

**Motivation:**

Tuberculosis, caused by *Mycobacterium tuberculosis*, remains a global health challenge driven by rising antibiotic resistance. Antimicrobial peptides offer a promising alternative due to membrane-disruptive activity and low resistance potential, yet the scarcity of TB-specific AMP data constrains targeted development. We present a reproducible deep learning protocol that integrates long short-term memory networks with transfer learning to classify and generate TB-active peptides.

**Results:**

Classifiers were pretrained on a large corpus of general AMPs and fine-tuned on curated TB-specific sequences using frozen encoder and full backpropagation strategies. We benchmarked four model variants [unidirectional and bidirectional long short-term memories (LSTMs), with and without attention] on a held-out TB test set; the unidirectional LSTM with a frozen encoder achieved the best performance (accuracy 90%, AUC 0.97). In parallel, LSTM-based generative models were trained to produce de novo TB-active peptides. A generator trained exclusively on TB data produced 94 of 100 peptides predicted as antimicrobial by AMP Scanner, outperforming transfer learning-based generators. Generated peptides were evaluated for antimicrobial activity, toxicity, structure, and AMP-like physicochemical traits, and four candidates shared ≥84% identity with known TB-AMPs.

**Availability and implementation:**

The complete model and data can be found at: https://github.com/linfeng-wang/TB-AMP-design.

## 1 Introduction

Tuberculosis (TB), caused by *Mycobacterium tuberculosis* (*Mtb*), remains a leading global health concern. Despite being preventable and treatable, TB continues to affect millions worldwide, with an estimated 10.8 million in 2023 alone (Global Tuberculosis Report 2024). This persistent burden is further compounded by the growing threat of drug-resistant TB strains. The emergence of multidrug-resistant and extensively drug-resistant TB (MDR/XDR-TB) significantly undermines current treatment regimens and public health responses. Multidrug-resistant tuberculosis (MDR-TB) refers to strains of *Mtb* that no longer respond to the two main first-line antibiotics, isoniazid (HR-TB) and rifampicin (RR-TB). In more severe cases, extensively drug-resistant TB (XDR-TB) arises, which not only resists these first-line drugs but also evades additional treatments, including key second-line antibiotics and newer drugs used as last-resort options (Meeting Report of the WHO Expert Consultation on the Definition of Extensively Drug-Resistant Tuberculosis 2020). Globally, an estimated 410 000 people developed MDR/RR-TB in 2022, with a treatment success rate of ∼60% (Global Tuberculosis Report 2024). This underscores the critical need for novel therapeutic strategies that can circumvent existing resistance mechanisms. The situation is further complicated by the presence of mixed infections (Wang *et al.* 2023), where patients harbour both drug-susceptible and drug-resistant strains, potentially masking resistance and contributing to treatment failure.

Against this backdrop, antimicrobial peptides (AMPs) have emerged as a promising class of therapeutics. These small, naturally occurring molecules, typically cationic and amphipathic, are key components of the innate immune system (Oliveira *et al.* 2021). By interacting with and disrupting microbial membranes, AMPs induce rapid microbial death. Some AMPs also act intracellularly, interfering with processes such as DNA replication, RNA transcription, or protein synthesis. Moreover, certain peptides contribute to immunomodulation by enhancing pathogen clearance through mechanisms such as autophagy induction. Their multifaceted modes of action, coupled with a lower propensity for resistance development, render AMPs highly attractive candidates for addressing the rising tide of drug-resistant TB (Oliveira *et al.* 2021).

In TB, the host’s ability to mount an effective AMP response is actively suppressed by *Mtb*. Recent studies have shown that the bacterial enzyme alanine dehydrogenase (Rv2780) depletes intracellular L-alanine, a metabolite that relieves inhibition on the NF-κB pathway via interaction with PRSS1 (Peng *et al.* 2024). By disrupting NF-κB activation, *Mtb* prevents the induction of key AMPs, including β-defensin 4 (DEFB4) (Peng *et al.* 2024), which plays a crucial role in limiting mycobacterial survival *in vivo*. These findings not only underscore the importance of AMPs in TB immunity but also highlight the need to identify novel peptides capable of bypassing or restoring these disrupted pathways. However, the pool of experimentally validated TB-active AMPs remains limited. This data scarcity poses a challenge for models to identify new targets, but can be addressed through transfer learning, a technique that allows models trained on large, general AMP datasets to be fine-tuned on smaller, TB-specific examples. By leveraging learned patterns of antimicrobial function, transfer learning enables the development of classifiers and generators tailored to *Mtb*, accelerating the search for novel therapeutic peptides in a context of growing drug resistance.

The field of AMP research has benefitted from the accumulation of extensive sequence data, with databases such as the Antimicrobial Peptide Database (APD) (Wang *et al.* 2016), Collection of Antimicrobial Peptides (CAMP) (Gawde *et al.* 2023), and the Database of Antimicrobial Activity and Structure of Peptides (DBAASP) (Pirtskhalava *et al.* 2021) These repositories encompass a diverse array of peptides, varying in length, structure, and activity spectra, providing a rich resource for computational analysis. Machine learning models can be trained to predict antimicrobial activity, classify peptides based on their properties, and even generate novel AMP candidates with desired characteristics (Wang *et al.* 2021). While AMP datasets specific to *Mtb* remain limited, the wealth of general AMP data is well-suited for machine learning approaches, which can uncover sequence-activity relationships and predict anti-mycobacterial potential. Models can be trained to classify peptide activity, identify host-pathogen interaction motifs, and generate new candidates tailored for TB. By leveraging these computational tools, we can begin to overcome the data scarcity that has historically constrained targeted TB-AMP development, accelerating the path towards novel therapeutics for drug-resistant TB.

Long short-term memory (LSTM) networks are a type of neural network specially designed to work with data that comes in sequences. Conventional neural networks have limited ability to capture long-range dependencies in sequential data, making it difficult to remember earlier inputs when separated by large temporal gaps. LSTMs solve this by using special ‘gates’ that control what information is remembered and what is forgotten as the sequence is processed (Al-Selwi *et al.* 2024). These gates act like switches that help the network focus on the most relevant parts of the data, even if they occurred far apart in the sequence. This makes LSTMs particularly useful in tasks where the order and context of information matter, such as language translation, predicting trends over time, or understanding patterns in biological data like DNA or protein sequences (Al-Selwi *et al.* 2024). Additionally, LSTM can predict the next element given a sequence of previous elements; hence, it has a sequence generative property by iteratively predicting the next element based on an expanding window.

A variety of computational tools have been developed for AMP prediction. Few TB-specific predictors such as AtbPpred (Manavalan *et al.* 2019) and iAtbP-Hyb-EnC (Akbar *et al.* 2021) have aimed to identify peptides active against *Mtb*. Additionally certain general predictors such as iAMPCN (Xu *et al.* 2023) have functional prediction including TB (Table 1, available as supplementary data at *Bioinformatics Advances* online). However, no existing approaches integrate transfer learning or generative frameworks for TB-specific AMP discovery. Our study builds upon this landscape by combining LSTM-based architectures with transfer learning to provide a unified classification-generation framework for TB-targeted AMPs.

In this study, we developed both classification and generative models based on LSTM architectures to explore the application of transfer learning in AMP research. Initially, we trained LSTM-based models on a comprehensive dataset of general AMP sequences, enabling the models to learn the underlying patterns and structural features associated with antimicrobial activity. Subsequently, we employed transfer learning to fine-tune these pre-trained models using a smaller, TB-AMP dataset. This approach allows the models to adapt their learned representations to the specific characteristics of AMPs effective against *Mtb*. By leveraging the knowledge acquired from the broader AMP dataset, the fine-tuned models can effectively classify and generate novel AMPs tailored to combat TB, addressing the pressing need for new therapeutics against drug-resistant strains.

## 2 Methods

### 2.1 Data

General AMP sequences (*n =* 31 029) were collected from several well-established databases, including CAMP (Gawde *et al.* 2023), DBAASP (Pirtskhalava *et al.* 2021), DRAMP (Kang *et al.* 2019), and APD (Wang *et al.* 2016). For TB-specific AMPs (TB-AMPs), 352 TB-specific AMP sequences were curated from AntiTBPDB (Usmani *et al.* 2018), GRAMPA (Witten and Witten 2019), DBAASP, DRAMP, and APD. To provide appropriate negative (control) examples for the general AMP classification task, two negative datasets were generated from UniProt using specific sequence filters. The first dataset was created using the following UniProt query: length: [10 TO 80] NOT antimicrobial NOT antibiotic NOT antiviral NOT antifungal NOT fungicide NOT secreted NOT secretory NOT excreted NOT effector NOT defensin AND annotation_score, designed to exclude proteins annotated with antimicrobial or secretory functions. The second dataset used a more constrained filter: length: [5 TO 30] NOT antimicrobial NOT antibiotic NOT antiviral NOT antifungal NOT fungicide NOT secreted NOT secretory NOT excreted NOT effector NOT defensin, to capture a broader representation of short, non-antimicrobial peptides for comparison with general AMPs.

For the TB-AMP classifier, 106 370 negative samples were similarly drawn from UniProt (The UniProt Consortium 2025) with careful matching of sequence length distributions to those of the positive TB-specific peptides. Three length-restricted subsets were created: short peptides (<20 residues), intermediate-length sequences (10–60 residues), and longer peptides (60–120 residues). Sequences overlapping with known AMP entries were excluded to prevent data leakage between classes.

All datasets underwent a standardized preprocessing pipeline. Sequences containing non-standard amino acids, ambiguous characters, or chemical modifications were removed. Peptides and sequences shorter than 10 residues or composed of single-residue repeats were filtered out to ensure biological relevance and diversity. All peptides were one-hot encoded using the 20 canonical amino acids as the encoding space, a format suitable for machine learning. No fixed sequence length was imposed; instead, sequences were dynamically zero-padded within each batch to match the longest sequence in that batch before being passed to the LSTM layers.

After all preprocessing steps, the final general AMP classification dataset consisted of 15 839 (/31 029; 51.0%) positive and 15 190 (/31 029; 49.0%) negative sequences. The processed TB-AMP classification dataset included 205 (/440; 46.6%) positive and 235 (/440; 53.4%) negative sequences.

To assess redundancy within the datasets, pairwise sequence identity was computed among all peptides using global alignment (Biopython pairwise2.align.globalxx). The identity for each sequence pair was calculated as the alignment score divided by the length of the longer sequence, expressed as a percentage. The resulting mean, median, and range of sequence similarities were used to quantify intra-dataset diversity. The positive TB-AMP dataset exhibited a mean pairwise identity of 21.3% (median 20.0%), while the negative dataset showed 20.1% (median 19.1%). These values indicate low redundancy and adequate sequence diversity across both classes, confirming that the datasets represent a broad and non-redundant peptide repertoire. For modelling training, training dataset, validation dataset and test dataset were generated from the full TB-AMP dataset. The test set consists of 89 sample (positive: 41, negative: 47).

For the generative task, TB-AMP sequences longer than 40 residues were segmented into non-overlapping fragments of 20 residues, while sequences of 40 residues or fewer were retained in full. Following length filtering and preprocessing, the final dataset used for TB-AMP classification and generation consisted of 245 TB-specific peptide sequences.

### 2.2 Classification model

**Figure 1.**
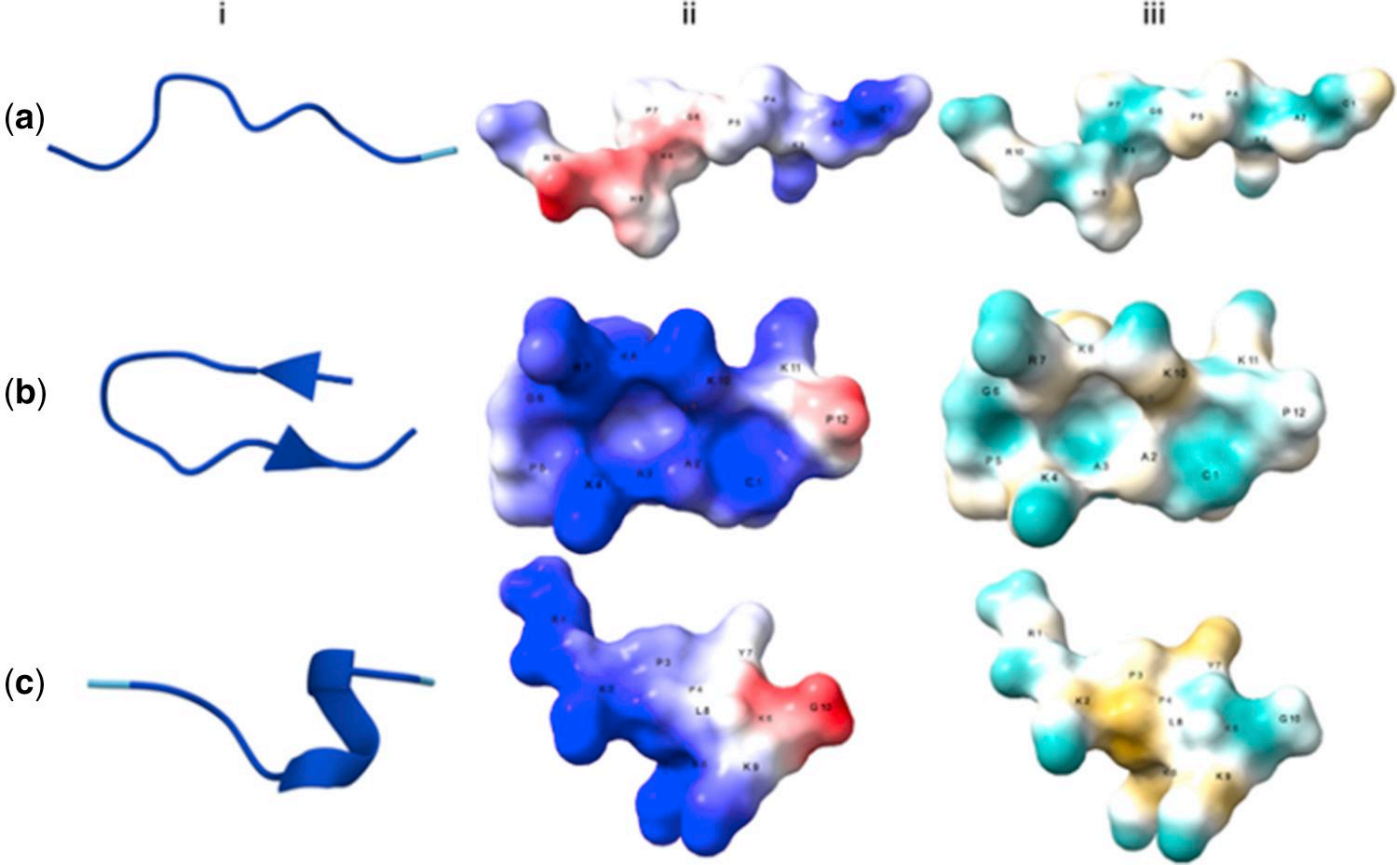
Generated protein structure prediction and visualization. Representative from each of the three structural classes—(a) linear, (b) β-turn, and (c) α-helix—was selected from the generated peptide set. For each peptide, the structure was predited using AlphaFold3 (i), and visualized in ChimeraX (Meng *et al.* 2023) for surface electrostatics and hydrophobicity. In panel (ii), surface colouring represents electrostatic potential (blue: positively charged; red: negatively charged). In panel (iii), hydrophobicity, cyan: hydrophilic regions; yellow: hydrophobic regions.

Building on previous work (Wang *et al.* 2021) that demonstrated the utility of LSTM models, we expanded the investigation by developing four distinct LSTM-based architectures to perform binary classification of AMP sequences using Pytorch (Paszke *et al.* 2019) (Fig. 1, available as supplementary data at *Bioinformatics Advances* online). The first was a standard unidirectional LSTM classifier with a single LSTM layer followed by dropout, a fully connected layer, and a sigmoid activation function. The second architecture used a bidirectional LSTM with outputs flattened across the time dimension before dropout and a final dense layer (BiLSTM with Flatten). The third architecture incorporated an attention mechanism into the unidirectional LSTM, applied across time steps before dropout and classification. The fourth, BiLSTM with Attention, combines bidirectional LSTM outputs with an attention mechanism, followed by dropout and sigmoid output. All models were trained using the Adam optimizer with a binary cross-entropy loss (BCELoss) function. Due to the stochastic nature of model training, each architecture was evaluated over 10 repeated runs of 25 epochs, and the mean and standard deviation (SD) of the performance metrics were reported (optimized model hyperparameters can be found in Table 2, available as supplementary data at *Bioinformatics Advances* online). For transfer learning, two strategies were explored: in the first, the pretrained LSTM layers were frozen and only the final layers were updated during fine-tuning (Frozen encoder); in the second, the pretrained weights were used to initialize the model, but all layers were updated through full backpropagation during fine-tuning (Full backprop). Umap (McInnes *et al.* 2020) were applied to the intermediate representation of the model to further assess and compare the models.

### 2.3 Generative model

The generative model in this study was constructed with a sequence-to-sequence architecture aimed at predicting the next amino acid in each peptide sequence. For instance, if the sequence GIGKFLHSAKKFGKAFV, GIGKFLHSAKKFGKAF is used as input and IGKFLHSAKKFGKAFV is used as target. Each input sequence is one-hot encoded and paired with its corresponding next-residue target sequence, allowing the model to be trained in an autoregressive fashion. During training, the model receives a sequence with the final residue removed and is tasked with predicting the subsequent amino acid at each position. All models were trained using the Adam optimizer, with categorical cross-entropy as the loss function, and evaluated using standard performance metrics including accuracy and receiver operating characteristic area under the curve (AUC). Two strategies of transfer learning, similar to the ones applied to classification, were also implemented. As output, sequences of varied lengths between 10 and 40 were generated. The temperature parameter was set to 1 to regulate sampling diversity. Various temperature values to generate peptides of 25 residues in length were tested and assessed according to AMP property using Antimicrobial Peptide Scanner vr.2 (Veltri *et al.* 2018) and percentage identity within the generated peptides and in comparison to the known TB-specific AMPs using pairwise2 algorithm. To evaluate the generative approach, each AMP and TB-AMP model trained using the transfer learning strategies, frozen layers and full backpropagation with pretrained weights as initialization was used to generate 100 peptide sequences. This number was chosen to balance exploratory diversity with downstream computational tractability, and is consistent with prior AMP generation studies, many of which use similar batch sizes (e.g. 100–200 peptides) for evaluation and screening purposes (Bolatchiev *et al.* 2022, Cao *et al.* 2025). To assess the model’s ability to rediscover known actives, 500 sequences were generated using the final TB-specific model and aligned against a curated reference set of TB-AMPs using global pairwise alignment via Biopython’s pairwise2 module.

### 2.4 Model tuning

Bayesian hyperparameter optimization for the classification models was conducted using the Optuna (Akiba *et al.* 2019) framework. A Bayesian optimization strategy was employed to tune key parameters, including the hidden dimension size (32–128), the number of LSTM layers (1–3), dropout rate (0.1–0.5), learning rate (0.0001–0.01), and weight decay (0.000001–0.01). For each model, the objective function was defined to maximize validation performance based on the area under the ROC curve (AUC) over ten training epochs. A total of 20 trials were executed for each optimization task. For transfer learning, the optimal hidden dimension and number of layers identified from training on the general AMP dataset were retained to ensure architectural consistency. The remaining parameters (dropout, learning rate, and weight decay) were re-optimized using Optuna (Akiba *et al.* 2019) under the same search space. This two-stage optimization approach was consistently applied across all classification model variants to ensure fair and comparable tuning.

### 2.5 Physiochemical and structural analysis

To further assess the quality and potential applicability of the generated peptide sequences, a series of external analyses were performed. The Antimicrobial Peptide Scanner v2 (Veltri *et al.* 2018) was used to independently verify whether each generated sequence was likely to possess antimicrobial activity. In addition, CSM-Toxin (Morozov *et al.* 2023) was employed to evaluate the toxicity profile of each peptide. Peptide hemolytic property classification was predicted using HermoPI2.0 with hybrid technique(ESM2-t6 + MERCI) using the default threshold value of 0.58 (Rathore *et al.* 2025). To select peptides with physicochemical properties [calculated using modlamp (Müller *et al.* 2017)] associated with antimicrobial activity, the following thresholds were applied: net charge ≥+2, hydrophobic moment >0.3, hydrophobicity between 0.2 and 0.6, and isoelectric point >7.5. These filters were chosen based on established AMP design principles to prioritize sequences with balanced amphipathicity. Finally, a similarity search was performed against known TB-AMPs using Blastp (Camacho *et al.* 2009). For three-dimensional structure prediction, AlphaFold3 (Abramson *et al.* 2024) was utilized to model the conformational properties of the selected sequences and visualized using XChimera (Meng et al. 2023).

### 2.6 Signed divergence word shift analysis

To compare the local sequence features between real and generated TB-AMPs, we conducted a signed divergence analysis of k-mer frequencies using a custom word shift approach. For each pair of peptide sets (e.g. known TB-AMPs vs. generated TB-AMPs), we extracted all overlapping k-mers (length = 2) and calculated their normalized frequency distributions with Laplace smoothing (ε = 1 × 10^−8^). For each k-mer, a symmetric, signed Kullback-Leibler (KL)-like divergence score was computed:


$$D(\mathrm{kmer})=\;\rho \cdot \log\frac{p}{q}-q\cdot \log\frac{q}{p}$$

where *p* and *q* represent the k-mer frequencies in the real and generated sets, respectively. This formulation captures both the magnitude and direction of divergence, allowing us to rank motifs by their contribution to distributional differences. The top 30 most divergent k-mers were visualized using horizontal bar plots, where positive values indicate enrichment in the known (real) AMPs and negative values indicate enrichment in the generated AMPs.

### 2.7 Jensen–Shannon divergence analysis

To assess the global dissimilarity between peptide sequence sets, we used scikit-learn (Pedregosa *et al.* 2011) to compute the Jensen–Shannon divergence (JSD) between k-mer distributions of each test set (known or generated TB AMPs) and two reference sets: general AMPs and non-AMP sequences. JSD was computed over normalized 2-mer frequency distributions using:


JSD(P∥Q)=12KL(P∥M)+12KL(Q∥M)where M= 12(P+Q).

This metric was selected for its symmetry, boundedness in [0,1], and robustness to zero counts. The resulting divergence scores quantify the extent to which the sequence composition of TB-AMPs deviates from general AMP patterns or random background sequences.

## 3 Results

### 3.1 Peptide classification

All LSTM-based architectures demonstrated robust classification capabilities, achieving high average accuracy and ROC-AUC scores when trained on the larger, general AMP dataset (Table 1). Among these, the BiLSTM architecture consistently delivered strong predictive performance across training regimes, with the frozen encoder approach achieving an average accuracy of 0.89 and AUC of 0.95. This suggests that the bidirectional nature of BiLSTM contributes to its capacity to capture key features even under transfer learning constraints.

**Table 1.** Long short-term memory (LSTM)-based model performance.^a^

Model	Training data	Accuracy (mean ± SD)	AUC (mean ± SD)	Sensitivity (mean ± SD)	Specificity (mean ± SD)
Vanilla LSTM	General AMP	0.7977 ± 0.0445	0.9381 ± 0.0174	0.9366 ± 0.0224	0.6766 ± 0.0730
	Frozen encoder	0.9045 ± 0.0056	0.9690 ± 0.0002	0.9244 ± 0.0073	0.8872 ± 0.0098
	Full backprop	0.8091 ± 0.0484	0.8649 ± 0.0548	0.7415 ± 0.0749	0.8681 ± 0.0886
	TB-AMP	0.8284 ± 0.0375	0.8861 ± 0.0218	0.7902 ± 0.0569	0.8617 ± 0.0803
BiLSTM	General AMP	0.8318 ± 0.0403	0.9490 ± 0.0199	0.9683 ± 0.0156	0.7128 ± 0.0757
	Frozen encoder	0.8920 ± 0.0205	0.9584 ± 0.0093	0.8195 ± 0.0569	0.9553 ± 0.0242
	Full backprop	0.8750 ± 0.0114	0.9441 ± 0.0071	0.8098 ± 0.0146	0.9319 ± 0.0208
	TB-AMP	0.8864 ± 0.0197	0.9553 ± 0.0085	0.8317 ± 0.0442	0.9340 ± 0.0242
LSTM + attention	General AMP	0.8591 ± 0.0211	0.9573 ± 0.0136	0.9390 ± 0.0366	0.7894 ± 0.0567
	Frozen encoder	0.5341 ± 0.0000	0.5960 ± 0.0725	0.0000 ± 0.0000	1.0000 ± 0.0000
	Full backprop	0.8273 ± 0.0396	0.9144 ± 0.0187	0.7780 ± 0.0941	0.8702 ± 0.1285
	TB-AMP	0.8261 ± 0.0449	0.9197 ± 0.0119	0.8098 ± 0.0937	0.8404 ± 0.1166
BiLSTM + attention	General AMP	0.8364 ± 0.0279	0.9597 ± 0.0118	0.9537 ± 0.0203	0.7340 ± 0.0515
	Frozen encoder	0.8102 ± 0.0536	0.8917 ± 0.0455	0.8707 ± 0.0994	0.7574 ± 0.1750
	Full backprop	0.8591 ± 0.0360	0.9257 ± 0.0158	0.8268 ± 0.0385	0.8872 ± 0.0767
	TB-AMP	0.8443 ± 0.1076	0.9256 ± 0.0487	0.7537 ± 0.2540	0.9234 ± 0.0701

a LSTM-based model performance (mean ± SD) trained on different training set and method: general AMP dataset, TB-AMP dataset, transfer learning with a frozen encoder, and transfer learning with full backpropagation. The models’ performance was all tested on the same TB-AMPs.

**Figure 2.**
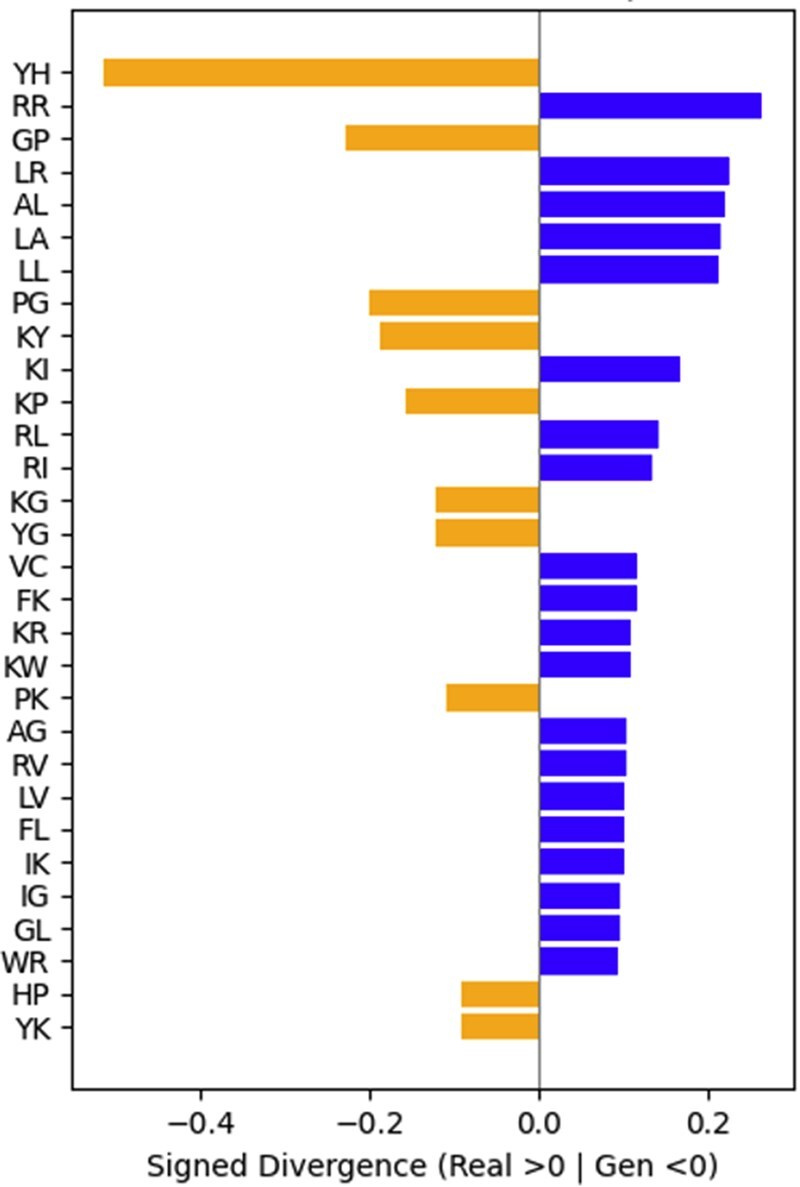
Signed KL divergence between 2mer for generated TB-AMP versus known TB-AMP. Signed divergence in 2-mer (dipeptide) frequencies between real TB-AMPs and generated TB-AMPs Divergence was computed using a symmetric KL-based measure for each k-mer, with positive values (blue bars) indicating enrichment in real AMPs and negative values (orange bars) indicating enrichment in generated AMPs. The top 30 most divergent 2-mers are shown.

Interestingly, the vanilla LSTM with a frozen encoder achieved the best overall performance (accuracy = 0.90, AUC = 0.97), demonstrating the greatest gain from transfer learning. This reinforces the utility of transfer learning when combined with selective weight freezing, allowing models to retain general AMP knowledge while fine-tuning task-specific parameters. This approach resulted in the most distinct clustering on intermediate representation in the model, with the highest silhouette score of 0.62 (Fig. 2, available as supplementary data at *Bioinformatics Advances* online). In contrast, attention-augmented LSTM variants showed more varied results. The BiLSTM with Attention model trained on the general dataset showed a consistent performance across datasets and transfer learning strategies for both general and TB-specific prediction. Whereas the simple LSTM had significant variability in sensitivity and specificity. Similarly, the LSTM with Attention model trained with a frozen encoder yielded the lowest accuracy overall (0.53), highlighting that attention mechanisms, while powerful, if without bidirectional context, may be more susceptible to overfitting or misalignment in low-data or domain-shifted scenarios.

### 3.2 AMP generation

Three generative models, derived from a Vanilla LSTM architecture, were employed to produce 100 peptide sequences each, with the temperature parameter set to 1 to control sampling diversity. The antimicrobial potential of these sequences was assessed using the Antimicrobial Peptide Scanner (Veltri *et al.* 2018), a tool designed to predict AMP activity based on sequence data. The model trained exclusively on TB-AMP data, without any transfer learning, yielded the highest number of sequences predicted as AMPs, with 94 out of 100 recognized. When utilizing pre-trained weights with full backpropagation across the entire model, 78 sequences were identified as AMPs. Conversely, the model that retained pre-trained weights while freezing the LSTM layers and updating only the final classification layer produced the lowest number of AMP predictions, identifying only 76 sequences as AMPs. These findings suggest that for generative purposes, training on domain-specific data without transfer learning may enhance the generation of sequences with desired antimicrobial properties.

**Figure 3.**
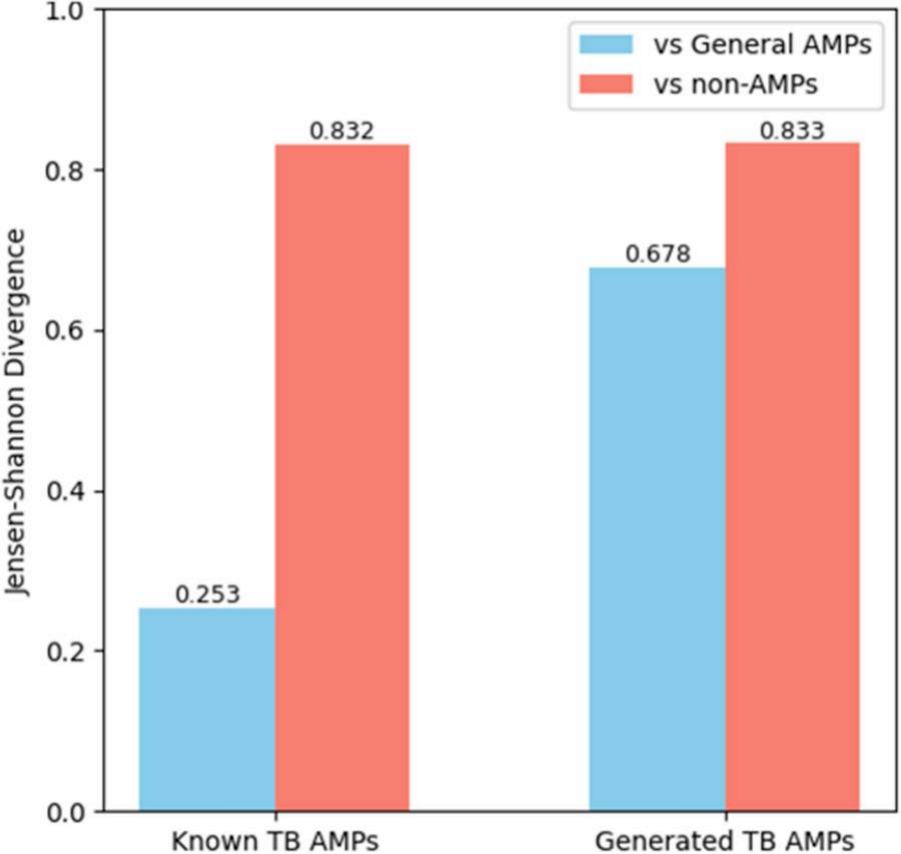
Divergence of TB-AMPs from general and non-AMPs.

To assess whether our model could rediscover previously characterized TB-active peptides, we generated 500 sequences using the TB-AMP fine-tuned model (temperature = 1) and compared them against a curated database of known TB-specific AMPs using global pairwise alignment. Four generated sequences showed ≥84% identity to established TB-AMPs. Different temperatures were also explored (Fig. 3, available as supplementary data at *Bioinformatics Advances* online). As expected, low temperatures increase the generation whereas the higher temperature increase the amino acid diversity.

### 3.3 Physiochemical and structural analysis

All 94 TB-AMPs generated were predicted to be non-toxic by CSM-Toxin (Morozov *et al.* 2023), whereas 11 were predicted to be hemolytic by HemoPI2.0 (Rathore *et al.* 2025). None matched existing entries in major databases, including APD, CAMP, DBAASP, DRAMP, and UniProt, confirming the novelty of the sequences produced by our models. This selection was further refined by applying general physicochemical filters characteristic of AMPs (Oliveira *et al.* 2021), resulting in a final set of 10 candidates enriched for AMP-like properties (Table 4, available as supplementary data at *Bioinformatics Advances* online). These filters included a net positive charge (≥+2) to favour electrostatic interactions with negatively charged bacterial membranes, and a hydrophobic moment (>0.3) indicative of amphipathic character essential for membrane insertion. An isoelectric point above 7.5 was used to ensure the peptides remain positively charged at physiological pH, while a Boman index below 10.5 kcal/mol was applied to reduce the likelihood of nonspecific protein binding and improve membrane selectivity. Together, these criteria enhance the likelihood that the selected candidates act through canonical AMP mechanisms.

The final candidates were then predicted to contain alpha helices, beta turns and linear structure by Alphafold3 (Abramson *et al.* 2024) (Fig. 1). Predicted α-helical segments in several candidates are consistent with a common AMP motif that aids membrane association. However, the mere presence of an α-helix does not imply antimicrobial activity, which arises from the combined balance of charge and amphipathicity and is also seen in non-helical scaffolds such as β-sheet defensins. A similarity search using Blastp (Camacho *et al.* 2009) against a curated TB-AMP database revealed that several *de novo* peptides do not share significant sequence identity with known TB AMPs, indicating that the generated sequences likely retain essential functional motifs while maintaining overall novelty.

### 3.4 Divergence analysis

Signed divergence analysis of 2-mer frequencies reveals distinct sequence preferences between known and generated TB-AMPs (Fig. 2). Motifs enriched in the real peptides (blue bars), such as RR, LR, AL, LA, LL, RL, FK, GL, are rich in A, L, and R, indicating classical AMP features like α-helicity, amphipathicity, and membrane affinity. In contrast, motifs enriched in the generated peptides (orange bars), including YH, GP, PG, KY, KP, YG, HP, contain residues like Y, P, and G, associated with kinks, bends, and structural flexibility. Pairwise sequence comparison between generated and known TB-AMPs showed a mean similarity of 47.3% (median 50.0%, range 40.0%–52.9%), These findings suggest that, while known TB-AMPs exhibit conserved structural patterns, the generative model introduces additional diversity that may reflect novel or enhanced functional characteristics relevant to TB-specific antimicrobial activity. This is supported by the observation that both generated and known TB-AMPs show a similar level of divergence from non-AMPs (JSD: 0.832–0.833; Fig. 3), indicating that the generated sequences retain AMP-like properties and are not random or non-functional. In contrast, when compared to general AMPs, known TB-AMPs show low divergence (JSD 0.253), reflecting strong overlap in sequence-level features. However, the generated TB-AMPs are more structurally distinct from general AMPs (JSD 0.678), potentially suggesting functional innovation or deviation from canonical AMP motifs.

## 4 Discussion

*Mtb* poses a major challenge due to its intrinsic resistance to conventional antibiotics. AMPs are promising alternatives, yet few have been validated against TB specifically. Notably, recent evidence highlights the functional importance of specific AMPs such as β-defensin 4, whose deletion impairs host defence against *Mtb in vivo*, supporting the feasibility of targeting TB with AMP-based strategies. To address this, we evaluated four LSTM-based architectures (LSTM, BiLSTM, LSTM with attention, and BiLSTM with attention) using both general and TB-AMP datasets, with and without transfer learning. All models performed well on the general AMP dataset, highlighting that sufficient data allows diverse architectures to learn effective sequence patterns. Under TB-specific conditions, performance varied more: BiLSTM and attention-based models generally outperformed unidirectional LSTM, benefitting from richer sequence context and positional focus.

Transfer learning had the most pronounced impact on the unidirectional LSTM, which gained nearly 10% in accuracy when using a frozen encoder. This likely reflects its simpler architecture, which benefits more from the introduction of informative pretrained representations. Additionally, the frozen encoder also limited additional variance introduced. In contrast, the bidirectional LSTM (BiLSTM), although more complex, showed less improvement, suggesting it is already capable of extracting relevant patterns without substantial reliance on pretraining. Application of transfer learning only increased variance of model, leading to overfitting. Interestingly, BiLSTM models with attention exhibited stable performance regardless of transfer learning, indicating that the additional contextual awareness from bidirectionality may contribute to robustness even in low-data settings. Meanwhile, attention-based models did not consistently benefit from transfer learning, likely due to their sensitivity to domain-specific patterns. Since attention mechanisms learn highly specialized focus weights, these may not transfer well between general AMP and TB-AMP domains, potentially misdirecting attention and reducing predictive accuracy. To optimize each model fairly, we allowed hyperparameters such as hidden dimension and number of LSTM layers to vary. While this adds complexity when comparing architectures directly, it ensures that each model operates at peak performance. This choice prioritizes practical accuracy over architectural uniformity; nonetheless, fixing these parameters across models remains a valid and potentially insightful alternative. Many AMP prediction tools exist, including Co-AMPpred (Singh *et al.* 2021) and AmPEP (Bhadra *et al.* 2018). However, these frameworks are trained on general AMP datasets and are not specific to *Mtb*. The existing method for TB-AMP prediction [iAtbP-Hyb-EnC (Akbar *et al.* 2021)] does not have generative capability. This gap underscores the value of domain-adapted architectures and motivates our transfer-learning and generative approach.

Compared to existing TB-specific classifiers, our best-performing model achieved 90% accuracy and 0.97 AUC, which is competitive with or exceeds current state-of-the-art predictors (Table 1, available as supplementary data at *Bioinformatics Advances* online). Unlike these prior methods, our NLP-based framework combines predictive accuracy with both interpretability and generative capability, enabling peptide *classification and design* within a unified architecture.

For the generative aspect, models trained directly on TB-AMPs outperformed those adapted through transfer learning, despite the smaller dataset. This highlights the importance of domain-specific learning for sequence generation, where pre-trained models may generate peptides that diverge from the structural or functional constraints of TB-AMPs. This likely reflects that transfer models pretrained on broad-spectrum AMPs capture general antimicrobial patterns but overlook TB-specific sequence biases. In contrast, the TB-only generator learns a compact, pathogen-focused distribution that still aligns with common AMP traits such as charge clustering and amphipathicity. Despite its smaller training set, it produced more confidently classifiable peptides, showing that domain-focused training can yield cleaner, more coherent representations.

The final generative models were run for 100 iterations, out of which 94 were determined to be non-toxic and potentially have antimicrobial properties. Through physicochemical profile filtering, nine peptide candidates were consistent with known AMP characteristics. Several of these sequences also showed partial similarity to TB-AMPs, suggesting potential pathogen specificity. Notably, k-mer divergence analysis revealed that generated peptides introduce more structural novelty relative to known TB AMPs while still retaining AMP-like features, as evidenced by their comparable divergence from non-AMPs and greater divergence from general AMPs. Furthermore, in an extended sampling of 500 generated peptides, four sequences shared over 84% sequence identity with known TB-AMPs. This demonstrates the model’s ability not only to generate novel candidates but also to recover biologically validated antimicrobial sequences, further supporting the model’s relevance to TB-specific drug discovery. While a preliminary toxicity screen indicated non-toxicity, further experimental studies are necessary to comprehensively assess their safety profiles. Overall, the model provided in the study is one of the only models with generative properties as well as classification using a NLP framework.

In addition, future work should focus on validating the antimicrobial efficacy of these candidates against *Mtb in vitro* and *in vivo*. Optimization strategies such as N- and C-terminal modifications or pharmacokinetic tuning could further enhance their therapeutic potential by improving serum half-life, bioavailability, and stability as AMP-based biologics. In the absence of such efforts, our work demonstrates the potential of machine learning approaches to uncover urgently needed therapeutic candidates for combating TB, a major global infectious disease.

## Supplementary Material

vbaf274_Supplementary_Data

## Data Availability

All datasets used in this study, including both general and TB-AMP sequences, as well as the negative control sets generated from UniProt, are available in the authors’ GitHub repository. The complete codebase for preprocessing, model training, evaluation, and sequence generation, including all LSTM-based architectures and transfer learning pipelines, is also provided. The repository ensures full reproducibility of the results presented in this study and can be accessed at: https://github.com/linfeng-wang/TB-AMP-design.
