# Supplementary material for: Long short-term memory-based deep learning model for the discovery of antimicrobial peptides targeting Mycobacterium tuberculosis
Source: Bioinform Adv. 2025 Oct 31;5(1):vbaf274. doi: 10.1093/bioadv/vbaf274 (PMC12603352; doi:10.1093/bioadv/vbaf274)
Supplement: vbaf274_Supplementary_Data [file vbaf274_supplementary_data.docx]

**SUPPLEMENTARY INFORMATION**

**S1 Figure**

**Overview of data preprocessing and model architecture**


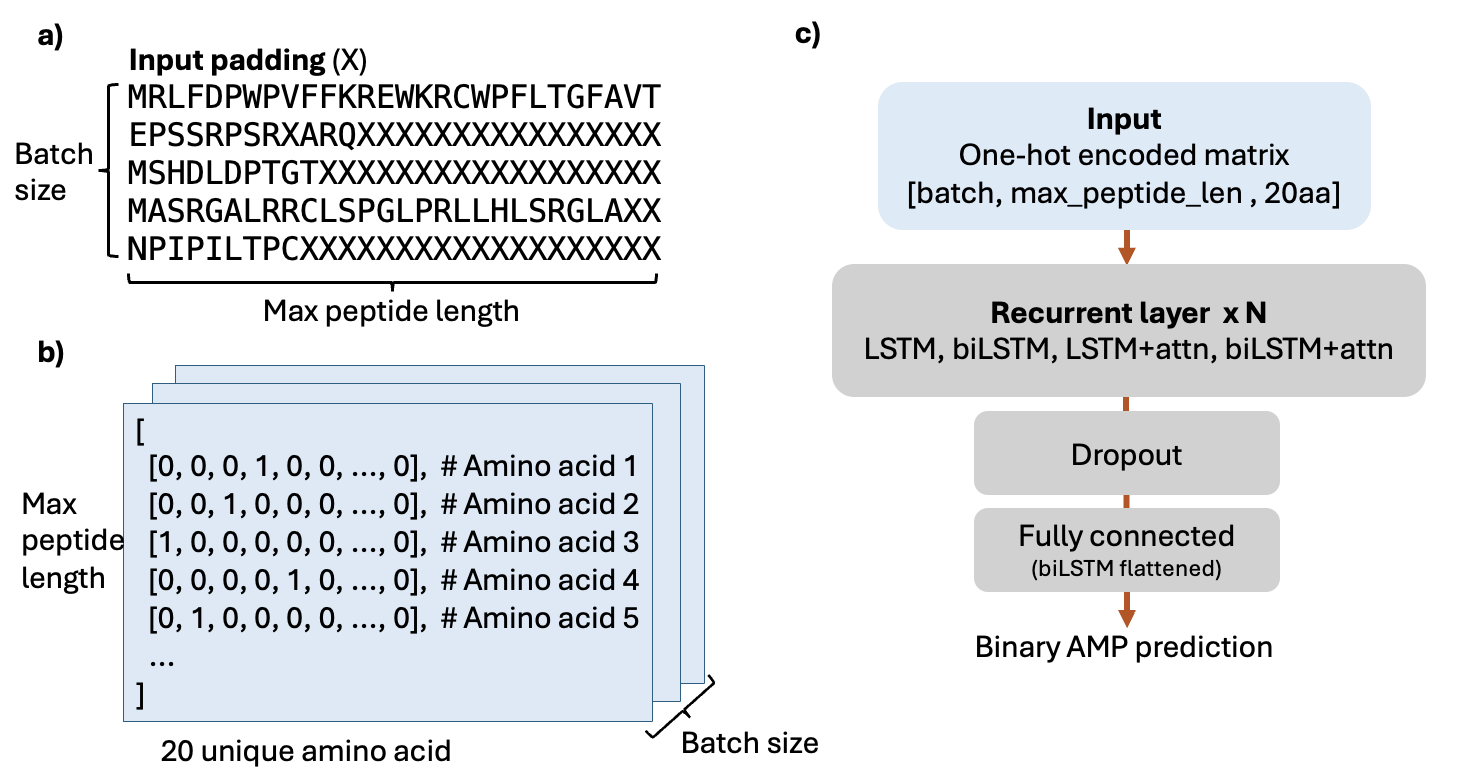


(a) Peptide sequences were padded with the character 'X' to match the maximum sequence length across the dataset. This ensured that all sequences in a batch shared the same length for efficient batching. (b) Each sequence was one-hot encoded into a binary matrix of shape [max peptide length × 20], where each row represents one amino acid out of 20 possible standard residues. These matrices were then stacked across the batch dimension, yielding a 3D tensor of shape [batch size, max peptide length, 20]. (c) The encoded input tensor was fed into recurrent neural network models. We evaluated four model variants: unidirectional LSTM, bidirectional LSTM (biLSTM), and both with attention (LSTM+attn, biLSTM+attn).

**S2 Figure**

**UMAP of visualisation of the model intermediate representations of TB-specific AMP**

**
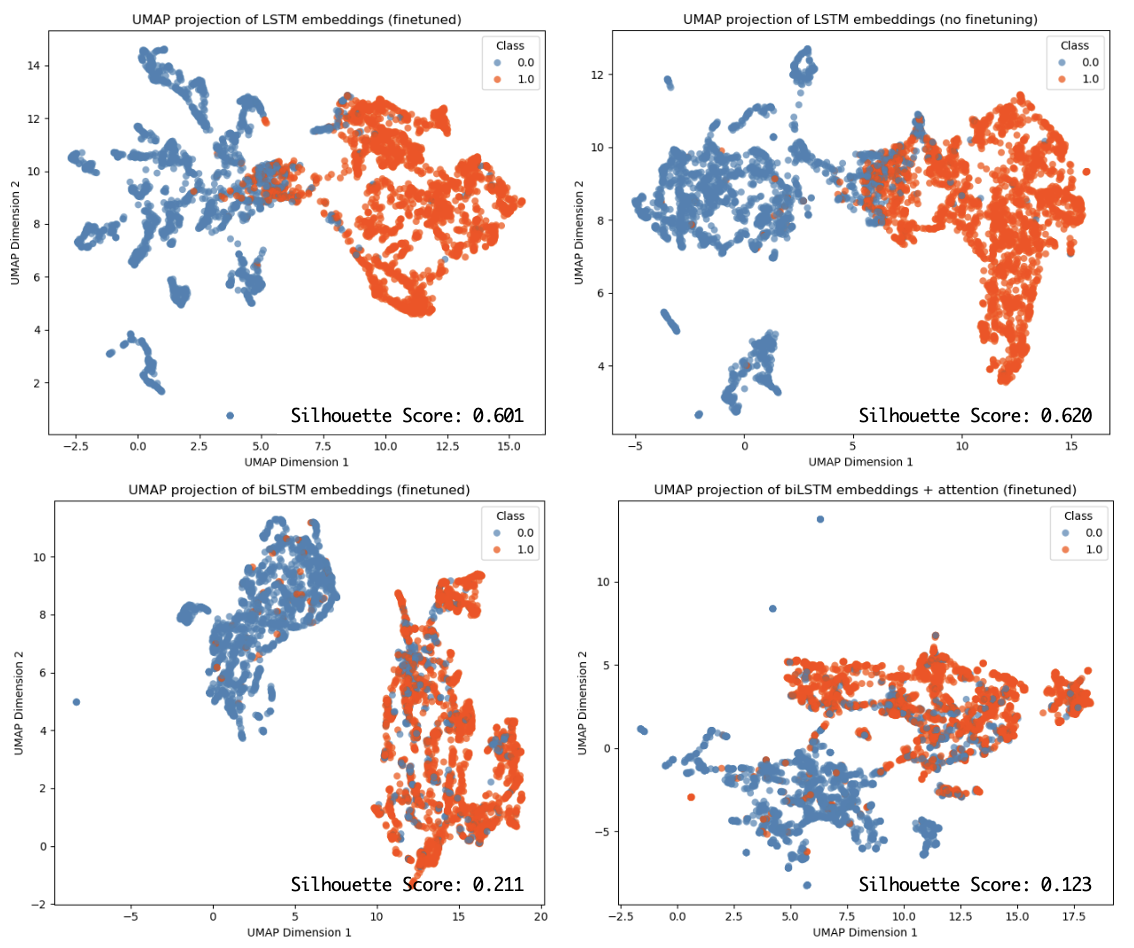
**

**S1 Table**

**Model comparison for TB-specific AMP prediction**

| **Tool Name** | **Type** | **Model Type / Algorithm** | **Performance*** | **Reference / Year** |
| --- | --- | --- | --- | --- |
| **AtbPpred** | TB-specific | Extremely randomized tree | Accuracy: 85.1%  AUC: 0.89 | Mabavalan et al., 2019^11^ |
| **iAntiTB** | TB-specific | Random forest, Support vector machine | Accuracy: 80-90%  AUC: 0.91-0.95 | Gautam et al., 2014^33^ |
| **iAtbP-Hyb-EnC** | TB-specific | Ensemble learning | Accuracy: 93% | Akbar et all., 2021^34^ |
| **iAMPCN** | Predicts 22 AMP classes incl. Anti-TB | Convolutional Neural Network | Accuracy: 77% (TB)  AUC: 0.89 | Xu et al., 2023^13^ |

*Performance as reported in the models’ publications

**S1 Table**

**Model comparison for TB-specific AMP prediction**

| **Tool Name** | **Type** | **Model Type / Algorithm** | **Performance*** | **Reference / Year** |
| --- | --- | --- | --- | --- |
| **AtbPpred** | TB-specific | Extremely randomized tree | Accuracy: 85.1%  AUC: 0.89 | Mabavalan et al., 2019^11^ |
| **iAntiTB** | TB-specific | Random forest, Support vector machine | Accuracy: 80-90%  AUC: 0.91-0.95 | Gautam et al., 2014^33^ |
| **iAtbP-Hyb-EnC** | TB-specific | Ensemble learning | Accuracy: 93% | Akbar et all., 2021^34^ |
| **iAMPCN** | Predicts 22 AMP classes incl. Anti-TB | Convolutional Neural Network | Accuracy: 77% (TB)  AUC: 0.89 | Xu et al., 2023^13^ |

*Performance as reported in the models’ publications

**S2 Table**

**Model hyperparameter from Bayesian optimisation**

| **Model** | **Training Data** | **Hidden dim** | **LSTM layers** | **Dropout** | **LR** | **L2 decay** |
| --- | --- | --- | --- | --- | --- | --- |
| **Vanilla LSTM** | General AMP | 95 | 1 | 0.203 | 0.004 | 4.00E-05 |
|  | Frozen encoder | - | - | 0.273 | 0.010 | 1.73E-03 |
|  | Full backprop | - | - | 0.454 | 0.003 | 7.05E-03 |
|  | TB-AMP | 82 | 1 | 0.408 | 0.005 | 4.49E-03 |
| **BiLSTM** | General AMP | 59 | 1 | 0.196 | 0.010 | 2.60E-05 |
|  | Frozen encoder | - | - | 0.386 | 0.010 | 1.52E-06 |
|  | Full backprop | - | - | 0.466 | 0.009 | 2.01E-05 |
|  | TB-AMP | 57 | 1 | 0.484 | 0.008 | 2.28E-03 |
| **LSTM + Attention** | General AMP | 85 | 1 | 0.455 | 0.006 | 1.23E-06 |
|  | Frozen encoder | - | - | 0.269 | 0.009 | 1.37E-06 |
|  | Full backprop | - | - | 0.467 | 0.002 | 1.05E-03 |
|  | TB-AMP | 110 | 2 | 0.246 | 0.005 | 9.18E-06 |
| **BiLSTM + Attention** | General AMP | 33 | 2 | 0.101 | 0.010 | 1.91E-05 |
|  | Frozen encoder | - | - | 0.355 | 0.002 | 2.74E-05 |
|  | Full backprop | - | - | 0.496 | 0.005 | 6.60E-04 |
|  | TB-AMP | 105 | 1 | 0.402 | 0.008 | 5.11E-06 |

LR: Learning rate, LSTM: Long-short term memory model

**S3 Table**

| **Temperature (T)** | **AMP Peptides** | **Mean Identity to Known (%)** | **Pairwise Mean Identity (%)** |
| --- | --- | --- | --- |
| 0.6 | 97 | 42.64 | 58.09 |
| 0.8 | 88 | 38.14 | 47.85 |
| 1 | 78 | 37.56 | 41.19 |
| 1.2 | 64 | 38.18 | 38.39 |
| 1.5 | 63 | 38.25 | 36.2 |

AMP peptides: the amount of generated peptides (out of 100) with a set length of 25 amino acid residues predicted to have AMP properties by Antimicrobial Peptide Scanner vr2.

**S4 Table**

**Physicochemical and structural properties**

| **ID** | **Sequence** | **Net Charge** | **pI** | **Hydrophobic Moment** | **Boman Index** | **Structural motifs** |
| --- | --- | --- | --- | --- | --- | --- |
| 11 | CAKPPGPHHR | 2.9 | 11.4 | 0.4 | 2.6 | Linear |
| 15 | IRYHKGPGKPHPGGYKGPRG | 6.1 | 11.5 | 0.3 | 2.3 | Linear |
| 38 | EIYRGHHRGPRYHYGYGKYG | 4.1 | 10.2 | 0.5 | 3.1 | Linear |
| 43 | CAAKPGRKLKKP | 5.8 | 11.8 | 0.6 | 2.2 | Beta turn |
| 44 | RAVYKHPKGGPP | 4.0 | 11.6 | 0.5 | 1.9 | Alpha helix |
| 59 | DFGPKGGPGYKKPYHPP | 3.0 | 10.3 | 0.3 | 1.4 | Beta turn |
| 73 | GAKYGGKYHKGGY | 4.0 | 10.4 | 0.4 | 1.2 | Linear |
| 77 | RKPPKKYLKG | 6.0 | 11.8 | 0.6 | 3.1 | Alpha helix |
| 97 | FPKYPRPGPYYG | 3.0 | 10.3 | 0.5 | 1.3 | Linear |

Physicochemical properties and structural predictions of selected *de novo* generated TB-AMPs. Net charge (favouring electrostatic interaction with bacterial membranes), isoelectric point (pI), hydrophobic moment (reflecting amphipathic character), Boman index (lower values suggest reduced nonspecific protein binding).
